# Concordance of Two Diabetes Diagnostic Criteria Using Fasting Plasma Glucose and Hemoglobin A1c: The Yuport Medical Checkup Centre Study

**DOI:** 10.1371/journal.pone.0047747

**Published:** 2012-10-17

**Authors:** Kazuo Inoue, Saori Kashima, Chisako Ohara, Masatoshi Matsumoto, Kimihiko Akimoto

**Affiliations:** 1 Department of Community Medicine, Chiba Medical Center, Teikyo University School of Medicine, Chiba, Japan; 2 Department of Public Health and Health Policy, Hiroshima University Institute of Biomedical & Health Sciences, Hiroshima, Japan; 3 Department of Public Health, Graduate School of Public Health, Teikyo University, Tokyo, Japan; 4 Department of Community-Based Medical System, Faculty of Medicine, Hiroshima University, Hiroshima, Japan; 5 Akimoto Occupational Health Consultant Office, Tokyo, Japan; Virgen Macarena University Hospital, Spain

## Abstract

**Background:**

We tested the concordance of the two diagnostic criteria for diabetes using fasting plasma glucose (FPG) and hemoglobin A1c (HbA1c) by the Japan Diabetes Society (JDS) and American Diabetes Association (ADA).

**Methods:**

We used data from 7,328 subjects without known diabetes who participated in a voluntary health checkup program at least twice between 1998 and 2006, at intervals ≤2 years. For repeat participants who attended the screening over two times, data from the first and second checkups were used for this study. At the first visit, diabetes was diagnosed both at FPG ≥7.0 mmol/L and HbA1c ≥6.5% using the JDS criteria. In addition, diabetes was diagnosed using two ADA criteria; ADA-FPG diabetes for persistent fasting hyperglycemia (FPG ≥7.0 mmol/L) or ADA-HbA1c diabetes for hyper-glycated hemoglominemia (HbA1c ≥6.5%), both at the first and second checkups. Subsequently, the concordance of diagnosis between the JDS and the ADA criteria was evaluated.

**Results:**

At the first checkup, 153 (2.1%) persons were diagnosed with diabetes by the JDS criteria. They had higher levels of risk factors for diabetes than non-diabetic subjects. Using the first and second checkups, 174 (2.4%) and 175 (2.4%) were diagnosed with diabetes by the ADA-FPG criteria, respectively. Among 153 subjects diagnosed with diabetes by the JDS criteria, 125 (81.7%) and 129 (84.3%) had ADA-FPG and ADA-HbA1c diabetes, respectively. The kappa coefficients of the JDS criteria with ADA-FPG and ADA-HbA1c criteria were 0.759 and 0.782 (*P*<0.001), respectively. In the subgroup analysis stratified by sex, the concordance was well preserved at the kappa coefficients around 0.8 (between 0.725 and 0.836).

**Conclusion:**

The JDS diagnostic criteria for diabetes have a substantial and acceptable concordance with the ADA criteria. The JDS criteria may be a practical method for diagnosing diabetes that maintains compatibility with the ADA criteria.

## Introduction

The diagnostic criteria of a disease are essential both for prevention and treatment of the particular disease. In 2010, the American Diabetes Association (ADA) and the Japan Diabetes Society (JDS) revised the diagnostic criteria for type 2 diabetes. The ADA adopted a repeated measure of either fasting plasma glucose (FPG) ≥7.0 mmol/l (persistent fasting hyperglycemia) or hemoglobin A1c (HbA1c) ≥6.5% (persistent hyper-glycated hemoglominemia) and did not adopt the simultaneous sampling of FPG and HbA1c [1]. In contrast, the JDS adopted a simultaneous test of FPG and HbA1c for the diagnosis of diabetes [2]. The JDS criteria is simple and convenient as it requires only one fasting blood sampling and may prevent the missing of a diagnosis due to the absence of a second test. On the other hand, the difference in the diagnostic criteria between the JDS and ADA generated the following issues: how are these two criteria in concord with each other and to what extent are these two criteria compatible with each other?

The difference between the JDS and ADA criteria appears to come from the difference in the two criteria’s ways of thinking about an early diagnosis and management of this disease. Both criteria value early diagnosis in order to increase clinical utility and convenience. The JDS criteria diagnose individuals as having diabetes in one day when those cases fulfill both the diagnostic range of FPG and HbA1c. The JDS values FPG more than HbA1c, and does not allow a diagnosis of diabetes with only HbA1c [2]. On the other hand, the ADA provides two options; either repeated FPG or repeated HbA1c samplings. The ADA does not recommend the mixed sampling of FPG and HbA1c, because these tests are not completely (100%) concordant, and thus can lead to confusion among clinicians [1].

Non-complete-concordance between the JDS and ADS criteria is self-obvious. What matters in practice is the compatibility of these different criteria as diagnostic tools. In addition, the diagnostic criteria of a certain disease should as much as possible be unified. When this is not the case in JDS and ADA criteria, their practical compatibility needs to be evaluated. Thus, we examined this question using a large sample from the Japanese population.

## Methods

### Study Subjects

**Figure 1:**
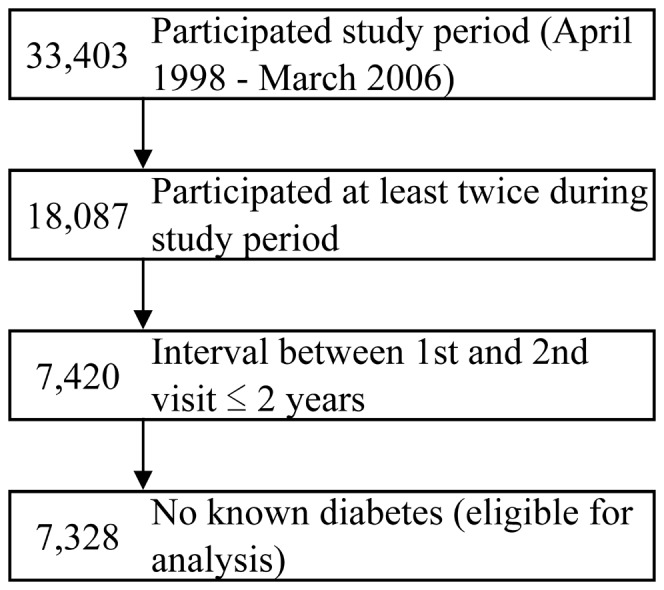
Identification of the 7,328 Study Subjects.

We used a dataset derived from the health screening program performed by the Yuport Medical Checkup Center in Tokyo, which has been described in our previous studies [3]–[5]. During the study period between April 1998 and March 2006, 34,303 persons voluntarily underwent a total of 97,365 checkups. Among these, 18,087 persons underwent evaluations at least twice during the study period. For those who had more than two evaluations during the study period, the data from the initial two checkups was used for this study. Among these, 7,420 persons underwent the second evaluation within two years of the first evaluation and this period would prevent an excessive time interval between two checkups. Further, 92 persons with known diabetes at the first checkup were excluded, and finally 7,328 persons were enrolled for this study (Figure 1).

All the evaluation procedures were performed in the same manner during the study period, including blood measurements. Height and weight were measured to calculate body mass index, which was defined as weight divided by height squared (kg/m^2^). Blood pressure was measured by trained nurses using a sphygmomanometer.

In accordance with the Private Information Protection Law, information that might identify subjects was safeguarded by the Medical Checkup Center. This study was approved by the review board of the Yuport Medical Checkup Center and a written informed consent for anonymous participation in epidemiological research was obtained at every evaluation.

### Laboratory Tests

A blood sample was obtained after overnight fasting and measured at the Center’s laboratory. For the measurements of FPG and HbA1c levels, a Toshiba TBA-40FR Autoanalyzer (Toshiba Medical Systems, Tokyo, Japan) was used. Plasma glucose level was measured via the hexokinase-G6PD method (Denka Seiken, Niigata, Japan) with an inter-assay coefficient of covariation of 3.0% or less. HbA1c level was measured by the latex immuno-agglutinin method (Determiner HbA1c, Kyowa Medex, Tokyo, Japan), with an inter-assay coefficient of covariation of 1.7–2.1%, which was comparable to that of plasma glucose and aligned to the JDS assigned values. The JDS value of HbA1c were converted into National Glycohemoglobin Standardization Program (NGSP) units in this study by adding 0.4% [2].

Other blood tests included serum levels of lipids and hepatic enzymes, and white blood cell count. Triglycerides, and total and high-density lipoprotein (HDL) cholesterol were measured using enzymatic methods (reagents supplied by Daiichi Pure Chemicals, Tokyo, Japan). Aspartate aminotransferase and alanine aminotransferase were measured using enzymatic methods (reagents supplied by Denka Seiken, Niigata, Japan), as were gamma-glutamyltranspeptidase levels (Wako Junyaku, Osaka, Japan). White blood cell count was measured using the differential count detection method (reagents supplied by Sysmex, Kobe, Japan).

### Diagnosis of Type 2 Diabetes via the JDS and ADA Criteria

At the first check-up, diabetes was diagnosed according to the JDS criteria; both for FPG ≥7.0 mmol/l and HbA1c ≥6.5% [2]. Using the first and second checkup data, diabetes was diagnosed according to the ADA criteria; either ADA-FPG diabetes (FPG ≥7.0 mmol/l both at the first and second checkup) or ADA-HbA1c diabetes (HbA1c ≥6.5% both at the first and second checkup) [1].

### Statistical Analysis

We examined the concordance of diabetes diagnosed by the JDS and ADA criteria. Particularly, we focused on how individuals diagnosed with diabetes using the JDS criteria could be identified by the ADA criteria. The kappa coefficients were calculated to evaluate the concordance among the JDS and ADA criteria. In general, kappa coefficients between 0.8 and 1.0 are interpreted as an almost perfect agreement, and those between 0.6 and 0.8 as a substantial agreement [6]. We also conducted a subgroup analysis stratified by sex. SPSS for Windows 19.0 (SPSS Inc., Tokyo, Japan) was used as the statistical software package. A cut-off *P* value <0.05 was used to determine statistical significance.

## Results

The duration between the first and second checkups was 1.17±0.35 (mean ± standard deviation, ranging from 0.16 to 2.0) years. At the first checkup, 153 (2.1%) subjects were diagnosed with diabetes by the JDS criteria. As shown in Table 1, they had higher levels of the risk factors for cardiovascular disease, such as high blood pressure, abnormal blood lipid levels, or being overweight, than the 7,175 non-diabetic subjects.

**Table 1:** Baseline characteristics of the 7,328 study subjects according to the presence of diabetes by the Japan Diabetes Society criteria.

		JDS criteria		
Variable	Total (*N* = 7,328)	Non diabetes (*N* = 7,175)	Diabetes (*N* = 153)	*P* value
Fasting plasma glucose_1_ (mmol/L)	5.40 (0.81)	5.32 (0.57)	8.95 (1.97)	<0.001
Fasting plasma glucose_2_ (mmol/L)	5.42 (0.83)	5.35 (0.62)	8.66 (2.02)	<0.001
Hemoglobin A1c_1_ (%)^a^	5.5 (0.6)	5.4 (0.4)	8.0 (1.5)	<0.001
Hemoglobin A1c_2_ (%)^a^	5.5 (0.6)	5.4 (0.5)	7.7 (1.4)	<0.001
Age (years)	54.0 (12.4)	54.0 (12.4)	58.5 (10.4)	<0.001
Male sex, n (%)	3,685 (50.3)	3,583 (49.9)	102 (66.7)	<0.001
Body mass index (kg/m^2^)	23.0 (3.1)	22.9 (3.1)	25.0 (3.2)	<0.001
Systolic blood pressure (mmHg)	124.1 (18.1)	123.9 (18.0)	135.1 (17.7)	<0.001
Diastolic blood pressure (mmHg)	74.9 (11.1)	74.7 (11.0)	80.7 (10.3)	0.010
Triglycerides (mmol/L)	1.06 (0.77–1.56)	1.06 (0.77–1.55)	1.57(1.07–2.03)	<0.001
Total cholesterol (mmol/L)	5.25 (0.90)	5.25 (0.90)	5.56 (0.91)	0.004
HDL cholesterol (mmol/L)	1.49 (0.39)	1.50 (0.39)	1.31 (0.33)	0.011
Asparate aminotransferase (U/L)	21 (18–25)	21 (18–25)	24 (20–32)	<0.001
Alanine aminotransferase (U/L)	18 (14–26)	18 (14–25)	25 (18–38)	<0.001
Gamma-glutamyltranspeptidase (U/L)	18 (11–32)	17 (11–32)	32 (18–50)	<0.001
White blood cell count (10^9^/l)	5.6 (4.7–6.6)	5.6 (4.7–6.6)	6.2 (5.3–7.4)	0.003

JDS, Japan Diabetes Society.

Data are expressed as mean (standard deviation), median (25 percentile–75 percentile) or number (%). Probability values are for comparison of categories of means (analysis of variance adjusted by sex and age) or percentages (chi-square test). The subscripts of 1 and 2 mean first and second visits, respectively.

a The JDS value of hemoglobin A1c were converted into National Glycohemoglobin Standardization Program units.

Table 2 shows the concordance between the JDS and ADA diabetes criteria. Using the first and second checkups, 174 (2.4%) and 175 (2.4%) were diagnosed as FPG diabetes and HbA1c diabetes according to the ADA criteria, respectively. Among 153 subjects diagnosed with diabetes by the JDS criteria, 125 (81.7%) and 129 (84.3%) fulfilled the ADA-FPG and ADA-HbA1c criteria of diabetes, respectively. Only 0.7% (49/7, 175) and 0.7% (46/7, 175) of the JDS non-diabetes were conversely identified with ADA-FPG and ADA-HbA1c diabetes, respectively. Although retrospective, 71.8% (125/174) and 73.7% (129/175) of the ADA-FPG or ADA-HbA1c diabetes were identified with diabetes in the JDS criteria.

**Table 2:** Concordance of diabetes diagnostic criteria between the JDS and ADA using FPG and HbA1c.

		ADA Criteria					
		FPG diabetes^a^			HbA1c diabetes^a^		
		Non diabetes (*N* = 7,154)	Diabetes (*N* = 174)	*K* (*P* value)	Non diabetes (*N* = 7,153)	Diabetes (*N* = 175)	*K* (*P* value)
JDS Criteria	Non diabetes (*N* = 7,175)	7,126 (99.3)	49 (0.7)	0.759 (<0.001)	7,129 (99.4)	46 (0.6)	0.782 (<0.001)
	Diabetes (*N* = 153)	28 (18.3)	125 (81.7)		24 (15.7)	129 (84.3)	

ADA, American Diabetes Association; FPG, fasting plasma glucose; HbA1c, hemoglobin A1c; JDS, Japan Diabetes Society; *K*, Kappa coefficient.

Data are expressed as number (percentage to the categories of the JDS criteria).

a The kappa coefficients between the ADA-FPG and ADA-HbA1c criteria was 0.668 (*P*<0.001).

The kappa coefficients between the JDS criteria and ADA-FPG or ADA-HbA1c criteria were 0.759 (*P*<0.001) and 0.782 (*P*<0.001), respectively. On the other hand, the kappa coefficient between the ADA-FPG and ADA-HbA1c criteria was 0.668 (*P*<0.001), which was lower than those between the JDS and ADA criteria.

Table 3 shows the results of the sex-stratified subgroup analysis. In men, the kappa coefficient between the JDS criteria and the ADA-FPG criteria slightly decreased to 0.725 (*P*<0.001). In general, the concordance was well preserved; the kappa coefficients stayed around 0.8 (0.725 and 0.835 for between the JDS criteria and the ADA-FPG criteria in men and women, and 0.779 and 0.782 for between the JDS criteria and the ADA-HbA1c criteria in men and women, respectively). In men, the JDS criteria were more concordant with the ADA-HbA1c criteria than the ADA-FPG criteria. In women, however, the JDS criteria were more concordant with the ADA-FPG criteria than the ADA-HbA1c criteria.

**Table 3:** Concordance of diabetes diagnostic criteria between the JDS and ADA using FPG and HbA1c stratified by sex.

		ADA Criteria					
		FPG diabetes^a^			HbA1c diabetes^a^		
		Non diabetes	Diabetes	*K* (*P* value)	Non diabetes	Diabetes	*K* (*P* value)
Men		(*N* = 3,558)	(*N* = 127)		(*N* = 3,573)	(*N* = 112)	
JDS Criteria	Non diabetes (*N* = 3,583)	3,540 (98.8)	43 (1.2)	0.725 (<0.001)	3,555 (99.2)	28 (0.8)	0.779 (<0.001)
	Diabetes (*N* = 102)	18 (17.6)	84 (82.4)		18 (17.6)	84 (82.4)	
Women		(*N* = 3,596)	(*N* = 47)		(*N* = 3,560)	(*N* = 63)	
JDS Criteria	Non diabetes (*N* = 3,592)	3,586 (99.8)	6 (0.2)	0.835 (<0.001)	3,574 (99.5)	18 (0.5)	0.786 (<0.001)
	Diabetes (*N* = 51)	10 (19.6)	41 (80.4)		6 (11.8)	45 (88.2)	

ADA, American Diabetes Association; FPG, fasting plasma glucose; HbA1c, hemoglobin A1c; JDS, Japan Diabetes Society; *K*, Kappa coefficient.

Data are expressed as number (percentage to the categories of the JDS criteria).

a The kappa coefficients between the ADA-FPG and ADA-HbA1c criteria was 0.632 (*P*<0.001) for men and 0.742 (*P*<0.001) for women.

## Discussion

More than 80% of the study subjects who were diagnosed with diabetes by the JDS criteria using the simultaneous sampling of FPG and HbA1c and were also diagnosed with diabetes using the ADA criteria using both a fasting hyperglycemia (FPG diabetes) and hyper-glycated hemoglominemia (HbA1c diabetes).

The concordance of the JDS and ADA criteria estimated by the kappa coefficients in all subjects was substantially good at between 0.76 and 0.78, nearly close to almost perfect agreement [6]. Even in the subgroup analysis stratified by sex, the kappa coefficients were well preserved at between 0.73 and 0.84. Although assumable, the kappa coefficients between the JDS criteria and either of FPG or HbA1c diabetes by the ADA criteria were better than that of the two criteria by the ADA (FPG diabetes and HbA1c diabetes). Thus, JDS criteria that only requires one day with morning fasting, may be a practical method for diagnosing diabetes that has acceptable concordance with the ADA criteria.

The features of this study include data by the same laboratory tests during the study period and a sufficient number of study subjects to examine the research question. There have been few studies that examined our research question, which addressed the compatibility of different diabetes criteria. If the compatibility was poor, the two criteria would identify a substantially different population to each other with diabetes, which would bring out clinical and public health concerns from the inconsistency in diagnostic criteria.

Some issues deserve to be mentioned as possible limitations. First, since the study subjects participated on a voluntary basis, they may be healthier than the general population, causing a selection bias. This would lead to a lower prevalence of diabetes than the more general population of Japan. In addition, 59 persons with known diabetes at the first checkup were excluded, and thus, the prevalence of diabetes in the studied sample would have been further reduced. Therefore, based on this study design, the prevalence of diabetes in this study sample may be recognized as that of ‘undiagnosed’ diabetes rather than the overall prevalence of diabetes. In this study, 153 (2.1%) persons were diagnosed as having diabetes by the JDS criteria. There has been no report in Japan on the prevalence of diabetes that is comparable with this study, however, the prevalence of undiagnosed diabetes was similar to two previous reports in other countries [7], [8]. There are an estimated 7.0 million persons with undiagnosed diabetes in the U.S. (2.2% of the whole population) [7]. Similarly, the prevalence of undiagnosed diabetes in the adult population of Manitoba was 2.2% in a Canadian study [8]. Second, there might be subjects who rapidly progressed to diabetes between the first and second checkups, who therefore were not eligible to participate in this health checkup hereafter. This would tend to cause another underestimation of the prevalence of diabetes at the second visit, and might cause a decrease in the observed concordance. Third, the JDS criteria appeared to have better concordance with the ADA HbA1c criteria than the ADA FPG criteria in men, and the opposite was the case in women. However, it is not clear whether or not this finding is caused by chance.

Type 2 diabetes can be diagnosed by other methods, for example through clinical signs of diabetes, and casual and post-load glucose levels [1], [2]. In clinical practice and for screening tests, however, FPG and HbA1c are the most likely to be used as diagnostic tools. This study indicated a good concordance between the JDS criteria (FPG and HbA1c in one encounter) and the ADA criteria (either of FPG or HbA1c over two encounters). The ADA criteria requires both initial and confirmatory testing to be performed with the same test (repeated FPG or repeated HbA1c samplings) to focus on the practicality of the diagnostic process [1]. But the simultaneous test of FPG and HbA1c advocated by the JDS requires only one fasting visit. If this test is sufficiently compatible with the ADA criteria whether the FPG or HbA1c test is used as shown in this study, the JDS and ADA methods may be compatible with each other at clinical levels.

FPG and HbA1c levels reflect different aspects of glucose metabolism. FPG levels largely depend upon insulin resistance and hepatic glucose production [9]. Postprandial plasma glucose levels are more closely correlated with HbA1c than FPG [10], and depend upon insulin resistance, hepatic glucose output and uptake, and the insulin secretion capacity of pancreatic beta cells [11], [12]. Accordingly, the combination of FPG and HbA1c cover a wider range of pathophysiological processes in diabetes than either alone, and thus have an advantage in the diagnosis of diabetes. In this sense, an adaption of HbA1c would be an advance in the diagnosis of diabetes, whether per the JDS or ADA criteria. The ADA criteria using the same test twice is expected to identify more persons with diabetes than the JDS test whether for FPG or HbA1c, which was observed in this study. Of note is that the concordance of the JDS criteria with either of the two ADA criteria of FPG or HbA1c diabetes was better than that between the two ADA criteria. That may additionally support the practical compatibility of the JDS and ADA criteria with each other.

OGTT is still considered as the gold standard for diagnosing diabetes, however, current clinical use of OGTT test has gradually declined due to time and cost considerations. Alternatively, FPG and HbA1c use has been increasing due to cost and time advantages. Only one sampling of FPG would not be accepted as a diagnosis of diabetes due to day-to-day variation [13], although a diabetes diagnosis is considered acceptable based upon a single fasting-glucose measurement for epidemiological estimates of diabetes prevalence and incidence [14], [15]. On the other hand, HbA1c well reflects mean blood glucose levels for 1–3 months, and such day-to-day variation is substantially lower than that of FPG [16].

The question of whether the observed substantial concordance can be generalized to the general population with a higher prevalence of diabetes has clinical importance. A population with a higher prevalence of diabetes naturally includes individuals with higher blood glucose and HbA1c levels than this study sample, so whether this concordance can be preserved among such a population is an issue. HbA1c and blood glucose levels were linearly correlated with each other even at high blood glucose levels [17], which leads to the assumption that the good concordance is maintained among a population with a higher prevalence of diabetes. Further studies that examine general populations need to be conducted in the future to address this issue.

The JDS has recommended a re-test within one month for all persons who are diagnosed as diabetic type by either only the FPG or HbA1c levels [2]. The ADA has not indicated a limited interval for re-test, although the ADA recommends an annual checkup for diabetes for individuals ≥45 years of age with risk for diabetes [18]. In general, intra-individual variation tends to increase with a longer duration between two tests, whether for FGP or HbA1c. On the contrary, the shorter the time interval between two administrations of a test, the less likely that changes will occur and the higher the reliability will be. Thus, this variation is likely to naturally decrease at the shorter period recommended by the JDS criteria than that of this study. Thus, a shorter period for re-examination than this study may lead to more concordance between the JDS and the ADA criteria than was observed in this study. Further evaluations are needed to confirm the study results are applicable for shorter time periods. When confirmed, the JDS diagnostic criteria for diabetes that only requires one day with morning fasting, may be a practical method for diagnosing diabetes that maintains compatibility with the ADA criteria.
